# Estimating the burden of scrub typhus: A systematic review

**DOI:** 10.1371/journal.pntd.0005838

**Published:** 2017-09-25

**Authors:** Ana Bonell, Yoel Lubell, Paul N. Newton, John A. Crump, Daniel H. Paris

**Affiliations:** 1 Oxford University Clinical Research Unit, National Hospital of Tropical Diseases, Hanoi, Vietnam; 2 Mahidol Oxford Tropical Medicine Research Unit, Mahidol University, Bangkok, Thailand; 3 Centre for Tropical Medicine and Global Health, Nuffield Department of Medicine, University of Oxford, Oxford, United Kingdom; 4 Lao-Oxford-Mahosot Hospital-Wellcome Trust Research Unit, Microbiology Laboratory, Mahosot Hospital, Vientiane, Laos; 5 Centre for International Health, University of Otago, Dunedin, New Zealand; 6 Department of Medicine, Swiss Tropical and Public Health Institute, Basel, Switzerland; 7 Faculty of Medicine, University Basel, Basel, Switzerland; University of California Davis, UNITED STATES

## Abstract

**Background:**

Scrub typhus is a vector-borne zoonotic disease that can be life-threatening. There are no licensed vaccines, or vector control efforts in place. Despite increasing awareness in endemic regions, the public health burden and global distribution of scrub typhus remains poorly known.

**Methods:**

We systematically reviewed all literature from public health records, fever studies and reports available on the Ovid MEDLINE, Embase Classic + Embase and EconLit databases, to estimate the burden of scrub typhus since the year 2000.

**Findings:**

In prospective fever studies from Asia, scrub typhus is a leading cause of treatable non-malarial febrile illness. Sero-epidemiological data also suggest that *Orientia tsutsugamushi* infection is common across Asia, with seroprevalence ranging from 9.3%–27.9% (median 22.2% IQR 18.6–25.7). A substantial apparent rise in minimum disease incidence (median 4.6/100,000/10 years, highest in China with 11.2/100,000/10 years) was reported through passive national surveillance systems in South Korea, Japan, China, and Thailand. Case fatality risks from areas of reduced drug-susceptibility are reported at 12.2% and 13.6% for South India and northern Thailand, respectively. Mortality reports vary widely around a median mortality of 6.0% for untreated and 1.4% for treated scrub typhus. Limited evidence suggests high mortality in complicated scrub typhus with CNS involvement (13.6% mortality), multi-organ dysfunction (24.1%) and high pregnancy miscarriage rates with poor neonatal outcomes.

**Interpretation:**

Scrub typhus appears to be a truly neglected tropical disease mainly affecting rural populations, but increasingly also metropolitan areas. Rising minimum incidence rates have been reported over the past 8–10 years from countries with an established surveillance system. A wider distribution of scrub typhus beyond Asia is likely, based on reports from South America and Africa. Unfortunately, the quality and quantity of the available data on scrub typhus epidemiology is currently too limited for any economical, mathematical modeling or mapping approaches.

## Author summary

Scrub typhus is a mite-transmitted infectious disease that can be life-threatening. Diagnosing this disease is difficult, requiring special techniques that are often not readily available. As the actual impact of scrub typhus on the population and its geographical distribution remains unknown, we searched systematically for available information in medical databases. Scrub typhus is common: more than every fifth person in areas where scrub typhus occurs carry antibodies as a sign of previous contact. All countries with an established surveillance system have recorded an increase in scrub typhus cases over the past 8–10 years, while reports from South America and Africa suggest a wider distribution beyond Asia. Scrub typhus is a serious disease: approximately 6% of cases die if untreated, and 1.5% if treated, but mortality can reach 13% in areas where the usual treatment does not always work well. Death rates of complications are higher, reaching 14% in brain infections, 24% with multiple organ failure, and pregnancies with scrub typhus can have poor outcomes, with high miscarriage rates. Despite many limitations on the amount and quality of available reports, we found that scrub typhus is a severely underappreciated tropical disease, affecting mainly rural populations, but increasingly urban areas as well.

## Introduction

Scrub typhus is an infectious disease caused by *Orientia tsutsugamushi*, an obligate intracellular bacteria, transmitted by the bites of chigger mites [1]. In Southeast Asia, scrub typhus is a leading cause of treatable non-malarial febrile illness [2]. The first accounts linking febrile illness with the appearance of “harmful” mites (Japanese: “tsutsuga” mushi) range back to 313 AD in China [3]. Scrub typhus was originally associated with the Asian-Pacific “Tsutsugamushi triangle,” until recent evidence from the Arabian Peninsula, Chile and possibly Kenya suggested a wider global distribution in tropical and subtropical regions [4–7].

The use of improved diagnostic methods, increased medical investigations and awareness have recently contributed to greater recognition of scrub typhus in some countries, such as in Laos, India, southern China, South Korea, and Japan [8]. There is also evidence suggesting that a combination of climate change and expansion of humans into previously uninhabited areas may play a role in both re-emergence and apparent rising incidence of scrub typhus [9–11].

There are no licensed vaccines for scrub typhus, and no systematic vector control efforts in place. Despite increasing awareness in endemic regions, the public health burden and global distribution of scrub typhus remains poorly known.

Although scrub typhus received much attention before and during the Second World War and to a lesser degree during the Vietnam/American war, basic epidemiology is poorly understood with limited data on incidence and burden of disease for patients, their families, societies and the economy. This ignorance is probably due to a combination of factors; clinical presentation is very similar to other causes of fever, diagnostic difficulties contribute to mis-diagnosis and under recognition, and appropriate diagnostic tests are not widely available. Following the discovery of chloramphenicol in the 1940s, the scientific interest dropped rapidly and scrub typhus has since received little global attention [12]. The data quoted by the World Health Organization (WHO) stating that over a billion people are at risk and one million cases are estimated per year is referenced to a paper published 20 years ago in 1997 [13, 14].

Extrapolation based on geographical mite distributions and densities are not helpful due to patchy data, limited by the dynamics of infected mite populations and insufficient characterization of transmitting vectors. With new data and improvements in approaches to estimating the burden of febrile illnesses, it is important to reevaluate the burden of scrub typhus.

Rationale for this study: Scrub typhus is among the leading causes of undifferentiated treatable fever in Asia. The mortality rates appear low at first glance, but considering the numbers of those exposed and/or infected a significant disease burden is expected globally. The following research questions were addressed: What is the estimated global burden of disease for scrub typhus? What data on seroprevalence and minimum incidence for scrub typhus are available by geographical regions? What data on DALYs, YLLs and YLDs are available, and what is the mortality rate of treated scrub typhus?

In this study we summarized the literature relating to the disease burden and economic impact of scrub typhus since the year 2000 in order to estimate the global incidence and burden of this disease.

## Methods

A literature search of three databases: Ovid MEDLINE (2000-present), Embase Classic + Embase (2000-present) and EconLit (2000-present) was conducted on 11^th^ April 2016 using three search strategies. First search terms: Scrub typhus, *Orientia tsutsugamushi*, *Rickettsia tsutsugamushi*, chigger borne rickettsiosis, chigger borne typhus, *Orientia tsutsugamushi* infection, *Rickettsia tsutsugamushi* infection, tsutsugamushi disease, tsutsugamushi fever (keyword) AND prevalence, incidence, epidemiology. A second search included the above search for scrub typhus and all variations AND cost, cost analysis, cost of illness, drug costs, economics, health care cost, hospital costs, cost benefit analysis, cost effectiveness analysis, quality adjusted life year. A third search included scrub typhus AND mortality or death on the 1^st^ Oct 2016, for which all currently available data was included (no date restrictions). Data on untreated mortality have been reported [15], and therefore only papers with treated infection were included in estimating mortality. All titles and abstracts were reviewed by 2 authors for inclusion and any disagreements were discussed and inclusion based on the senior author’s opinion. Only English language publications were included.

**Fig 1:**
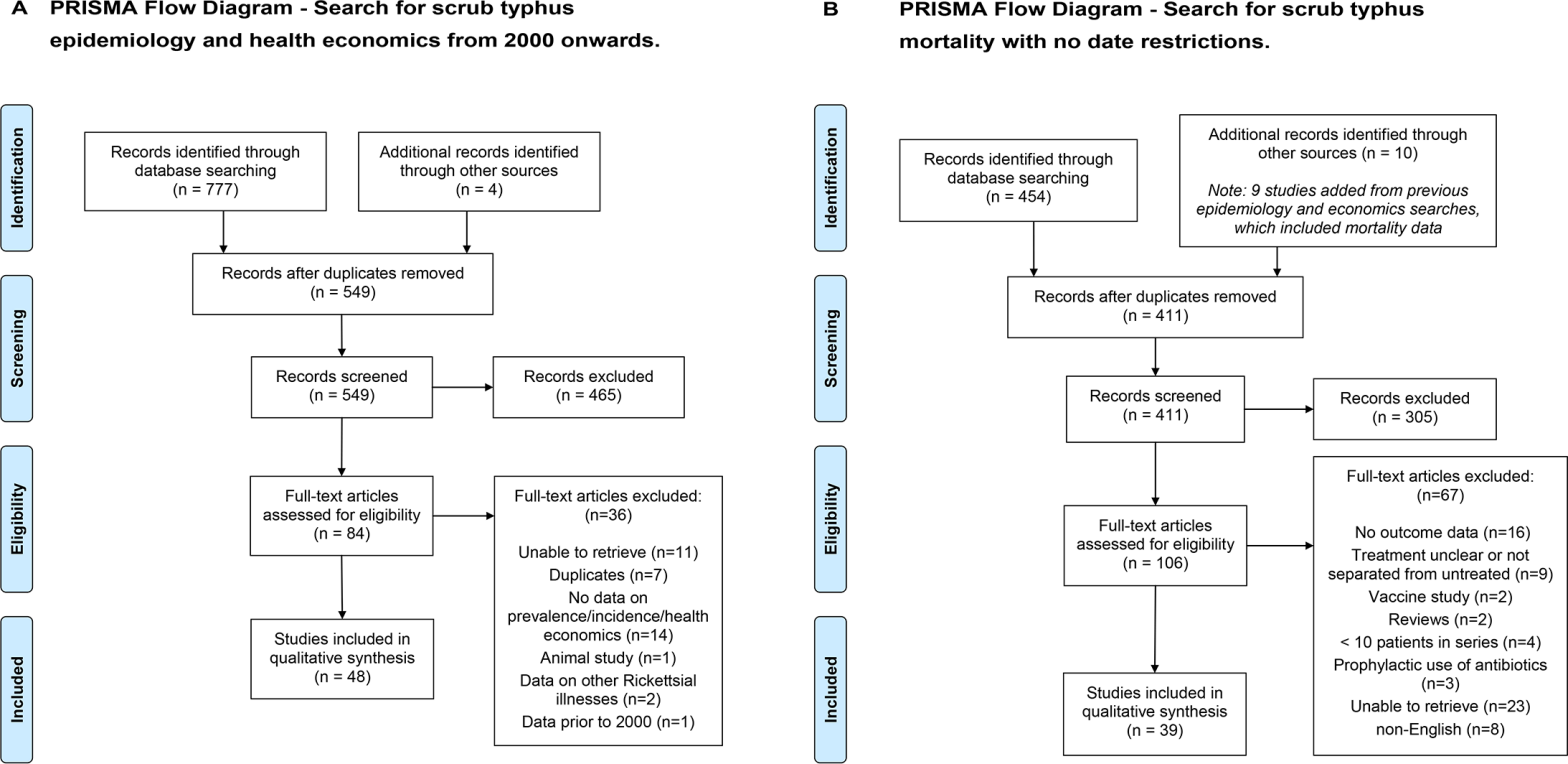
PRISM flow diagrams summarizing the search results. Panel A, search for scrub typhus epidemiology and health economics from 2000 onwards. Panel B, search for scrub typhus mortality with no date restrictions. Flow diagrams were downloaded from www.prisma-statement.org.

A total of 190 publications were selected for full article review (Fig 1A and 1B, S1 File). The final number of articles included for full data extraction was 87. The data extraction form was trialed on the first 5 papers and required minor alterations. Due to the limited nature of data available no summary measures were applied. Studies were examined for selection bias and graded as follows:

1. Population based active surveillance / community surveillance / hospital surveillance.
2. Prospective, consecutive patient case series with no inappropriate exclusions / retrospective case series / non-consecutive case series / reference laboratory series.
3. Exclusion of patients (i.e. most unwell patients treated) likely to significantly affect outcome / summary of case reports from literature.

Papers were also graded on diagnostic tests used:

1. Pathogen detection through cell culture/animal inoculation. PCR positivity. IFA serological diagnosis with either sero-conversion or fourfold antibody response.
2. Single high Weil Felix titre of ≥1:100 for all samples / Single high IFA titre >1:50 for all samples / IgM or IgG ELISA positive.
3. Single Weil Felix or IFA titre, non-significant for all patients / No record of significant Weil Felix or IFA titre / Clinical diagnosis / unspecified.

## Results

Of the 87 studies included, 44 (50.6%) gave information on incidence, seroprevalence and/or prevalence in febrile inpatients (denominator = febrile cases per year), whilst health economic or burden of disease data were given in 4 studies (4.6%) and mortality data in 38 (43.7%). The publications with no apparent patient selection bias and use of grade A evidence to diagnose scrub typhus were few (16/87, 18.4%). A total of 143,544 patients with scrub typhus were described in the included papers. Females were reported to be more commonly infected than males 77,204 versus 57,535 (57.3% versus 42.7%, respectively).

### Incidence surveillance data

Five countries report a passive national surveillance system for scrub typhus.

In South Korea scrub typhus was designated a group III notifiable disease (requiring mandatory reporting and routine monitoring) in 1994. Cases are confirmed by the Korean Centre for Disease Control and Prevention (KCDC) and must show one of the following: an increase in the IFA IgM to *O*. *tsutsugamushi* of ≥ 1:16; an increase in the anti-*O*. *tsutsugamushi* IFA IgG titre to ≥1:256; a ≥ 4 fold increase in IFA titre. Data from KCDC suggest that the annual minimum incidence increased from 5.7 to 17.7/100,000 people from 2001 to 2012 (>3-fold) (Table 1) [16–18]. Interestingly, the number of patients recorded in urban areas has also increased dramatically, for example, the annual minimum incidence in Ulsan Metropolitan City increased from 2.8/100,000 in 2003 to 59.7/100,000 in 2013 (>21 fold). In Seoul there is evidence of urban scrub typhus, further demonstrating the changing geographical scope and habitat of infected chigger mites [16].

**Table 1:** Estimates of incidence and sero-prevalence per country.

**First Author**	**Country**	**Incidence/100,000**	**Year data collected**	**Total number infected**	**Patient Selection Grade**	**Diagnostic Grade**
Park	South Korea	17.7	2012	8,604	1	A
Yasunaga	Japan	3.6	2007–2008	210	3	A
Wu	China	1.22	2014	16,050	1	A
Lee	Taiwan	14.3	2000–2004	1,396	1	A
N/A	Thailand	11.8	2015	7,696	1	A
**First Author**	**Country, Region**	**Sero-prevalence**	**Year data collected**	**Total number tested**	**Patient Selection Grade**	**Seroprevalence Diagnostic Grade**
Maude	Bangladesh	23.7%	2010	1,209	1	B
Richards	Indonesia, Gag Island	9.3%	2003	53	2	B
Vallée	Laos, Vientiane	20.7%	2006	2,002	1	B
Tay	Malaysia, Western Malaysia	17.9%	2007–2010	280	2	B
Spicer	Papua New Guinea	27.9%	2001	140	2	B
Premaratna	Sri Lanka	26.3%	2008	57	3	B

Patient selection grade: Grade I = Population based active surveillance / community surveillance / hospital surveillance, Grade II = Prospective, consecutive patient case series with no inappropriate exclusions / non-consecutive case series / retrospective case series / reference laboratory series, Grade III = Exclusion of patients (i.e. most unwell patients treated) likely to significantly affect outcome / summary of case reports from literature.Diagnostic grade: Grade A = Pathogen detection through cell culture/animal inoculation. PCR positivity. IFA serological diagnosis with either sero-conversion or fourfold antibody response, Grade B = single sample with high titre/ELISA IgG/IgM, Grade C = Single high Weil Felix or IFA titre but titre non-significant for all patients / No record of significant Weil Felix or IFA titre / clinical diagnosis / unspecified.

In Japan scrub typhus is a notifiable disease and must be reported to the National Epidemiological Surveillance of Infectious Diseases (NESID) within 7 days of diagnosis by a physician. Confirmed cases are based on: isolation or identification of the organism in the blood; PCR positivity; detection of serum IgM; a ≥ 4 fold increase in IFA titre. Data from NESID show an increase of annual minimum incidence from 0.6/100,000 in 2000 to 3.6/100,000 in 2008 (6-fold) [19, 20].

In Thailand, scrub typhus patients have been reported to the Bureau of Epidemiology for the last 30 years. Data can be viewed online on the homepage available under URL: http://www.boe.moph.go.th/boedb/surdata/disease.php?dcontent=situation&ds=44

Cases are defined based on one or more of the following: isolation or identification of the organism in the blood or tissue sample; PCR positivity; a ≥ 4 fold increase in IFA titre (IgG and/or IgM); ≥ 1:400 IFA in acute serum (IgG and/or IgM); IgM ELISA positivity. Data from the Bureau of Epidemiology noted an increase of annual minimum incidence from 6.0/100,000 in 2003 to 17.1/100,000 in 2013 (2.9 fold) [21].

In China, scrub typhus is a notifiable disease that must be reported to the China Center for Disease Control and Prevention. Cases are defined as those with clinically compatible infection and one or more of the following; isolation or identification of the organism in a blood or tissue sample; PCR positivity; a ≥ 1:160 Weil-Felix test; a ≥ 4 fold increase in IFA titre (IgG and/or IgM). The reported countrywide minimum incidence increased from 0.1/100,000 to 1.1/100,000 people/year from 2006 to 2014 (>11-fold) [22]. The reported incidence rates vary widely by region with the southern provinces more affected. Guangdong Province saw an increase in reported annual minimum incidence from 0.4/100,000 to 3.6/100,000 people from 2006 to 2013 (>8-fold), whereas in 2012 the provinces of Laiwu and Guangzhou City had annual incidences of 5.5/100,000 and 9.9/100,000 people, respectively [10, 23–25].

### Seroprevalence data

There are seroprevalence data available from Bangladesh, Indonesia, Laos, Malaysia, Papua New Guinea and Sri Lanka (Table 1). Seropositivity ranged from 9.3%–27.9% suggesting high background exposure levels to *O*. *tsutsugamushi* in these countries [26–31]

### Fever studies

There are several case series describing the frequency of scrub typhus among patients presenting with fever. In India, scrub typhus was the causative agent in 16.1–96.9% of febrile patients presenting to hospitals (Table 2). However, these studies all suffer from selection bias, as other causes of febrile illness had already been excluded. Studies from Cambodia, Laos, Nepal, and Kenya were subject to less bias as they included complete prospective series of patients presenting with fever to healthcare facilities and demonstrated rates from 1.8–22.3% (Table 2).

**Table 2:** Data on epidemiology of scrub typhus from fever studies (hospital based incidence).

First Author	Country, region	Year	Patients with scrub typhus n (%)	Total patients in study	Patient selection grade	Diagnostic Grade
Roopa [40]	India, Pondicherry	2012–2015	225 (41.3%)	545	2	B
Borkakoty [41]	India, Arunachal Pradesh	2013	31 (96.9%)	32	3	B
Kumar [38]	India, Chandigarh	2011–2012	49 (24.4%)	201	2	A
Narvenkar [42]	India, Goa	2009–2010	15 (34.1%)	44	2	B
Chrispal [43, 44]	India, Vellore	2007–2008	189 (47.5%)	398	2	B
Kamarasu [45]	India, Tamil Nadu	2004–2005	204 (16.1%)	1,270	2	B
Sharma [46]	India, Himachal Pradesh	2003–2004	52 (34.7%)	150	2	B
Vaz [47]	India, Jammu	2002	12 (50.0%)	24	2	B
Mueller [48]	Cambodia, remote Western and Eastern provinces	2008–2010	54 (3.7%)	1,475	2	A
Kasper [49]	Cambodia, within 50km Phnom Phenh	2006–2009	35 (1.8%)	1,906	2	A
Mayxay [50]	Laos, northwest Laos and southern Laos	2008–2010	122 (6.5%)	1,938	2	A
Blacksell [51]	Nepal, Kathmandu	2002–2004	23 (22.3%)	103	2	A
Reller [52]	Sri Lanka, southern	2007	9 (1.0%)	883	2	A
Susilawati [53]	Australia, Cairns	2008–2011	2 (0.6%)	340	2	A
Thiga [54]	Kenya, 6 regions	2015	76 (4.8%)	1,401	2	B

Patient selection grade: Grade I = Population based active surveillance / community surveillance / hospital surveillance, Grade II = Prospective, consecutive patient case series with no inappropriate exclusions / non-consecutive case series / retrospective case series / reference laboratory series, Grade III = Exclusion of patients (i.e. most unwell patients treated) likely to significantly affect outcome / Summary of case reports from literature. Diagnostic grade: Grade A = Pathogen detection through cell culture/animal inoculation. PCR positivity. IFA serological diagnosis with either sero-conversion or fourfold antibody response, Grade B = single sample with high titre/ELISA IgG/IgM, Grade C = Single high Weil Felix or IFA titre but titre non-significant for all patients / No record of significant Weil Felix or IFA titre / clinical diagnosis / unspecified.

Data from specific sub-populations are presented in Table 3. Two studies describe the importance of scrub typhus in women during pregnancy from Laos and the Thai-Myanmar border—with scrub typhus occurring in 3.6–5.4% of febrile patients [32–34]. Maternal infection with scrub typhus during pregnancy was associated with poor maternal and fetal outcomes; 2/9 (22.2%) of cases in Laos and 4/11 (36.4%) in Thailand/Myanmar suffered either abortion or stillbirth.

**Table 3:** Publications investigating scrub typhus affected specific sub-populations.

First Author	Country	Year	Population specifics	Number ST (%)	Diagnostic Grade
Dittrich *et al*. [35]	Laos, Vientiane	2003–2011	Patients enrolled if admitted with CNS infections and lumbar puncture indicated	31/1051 (2.9%)	A
Chansamouth *et al*. [34]	Laos, Vientiane,	2006–2010	All febrile pregnant inpatients	9/250 (3.6%)	A
McGready *et al*. [32]	Thai-Burmese border	2004–2006	All febrile pregnant patients	11/203 (5.4%)	A
Premanatna *et al*. [29]	Sri Lanka	2008	Consecutive admission of military personnel with fever	26/49 (53%)	B
Nadjm *et al*. [55]	Vietnam, northern Vietnam	2001–2003	Consecutive patients admitted to national referral hospital of infectious diseases, with no immediate diagnosis	251/7226 (3.5%)	B

Among Lao patients with meningitis/encephalitis, 16.0% of those with a diagnosed bacterial cause for their infection had evidence for scrub typhus [35]. However, only 54.8% of these patients received treatment with appropriate antimicrobials during admission and the mortality rate associated with CNS complications was 13.6%. There are no data on morbidity or long-term sequelae available.

### At risk population

National surveillance data from patients in China, Japan, Korea and Taiwan suggest that the age group of 60–69 years was at highest risk of scrub typhus [18, 20, 22, 36]. In Thailand those aged 45–54 years were most commonly infected. In Japan and Thailand males were more at risk of scrub typhus but in all other countries with reports, females are more at risk. In South Korea, China, Taiwan and Thailand farmers were most at risk (38,183/54,558–70% of infections in China from 2006–2014); unfortunately such data are lacking from Japan. Age stratification in untreated mortality revealed increasing risk with increasing age, with the age classes 51–60 and >60 years old associated with a 45.6% and 59.8% mortality rate respectively [15].

### Complications and sequelae

The long-term impact of infection with scrub typhus has barely been examined. In Taiwan the hazard ratio of developing acute coronary syndrome was 1.4 (95% CI 1.1–1.8) in those with previous infection with scrub typhus compared to the general population without [37]. A recent case series from India that included patients with unexplained fever and/or multi-system involvement, found 24.4% to have scrub typhus, and 53.1% of patients with scrub typhus had acute kidney injury [38]. A retrospective cohort of severe scrub typhus cases admitted to an ICU in South India, found that respiratory complications requiring mechanical ventilation occurred in 87.9%, and that dysfunction of 3 or more organ systems occurred in 85.2% [39].

### Case fatality ratios

**Fig 2:**
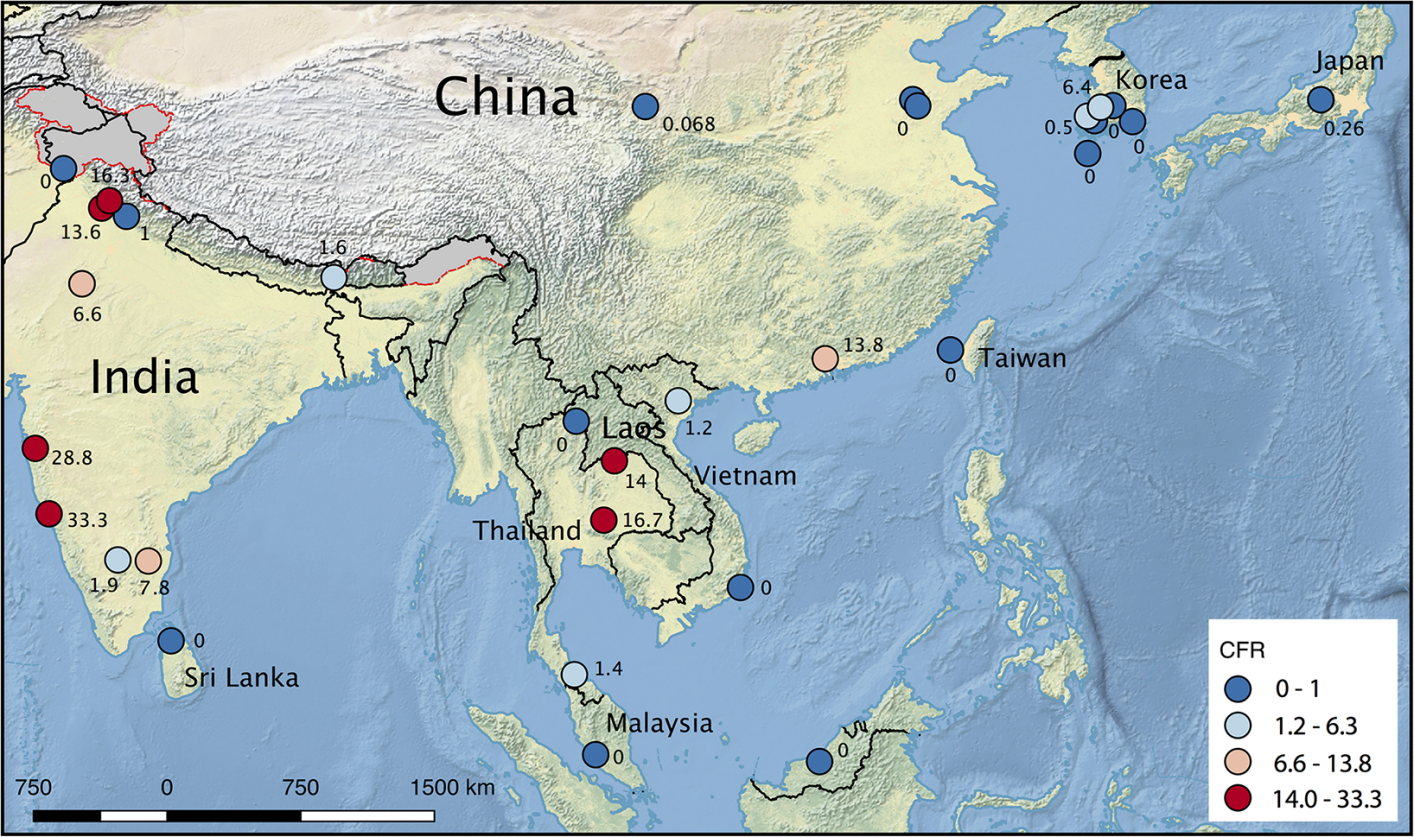
Case fatality (%) of scrub typhus reported in published case series and studies. This figure shows the locations from included case series and study reports in a map; the case fatality (%) reported is color coded (blue 0–1%; pale blue 1.2–6.3%; pale red 6.6–13.8%; and red 14–33.3%), and the detailed data of the studies included is summarized in Table 4. Source of map: http://www.naturalearthdata.com, accessed on the 14th July 2017. Kashmir and Arunachal Pradesh are depicted in grey with borders as red dashes, due to either disputed or indeterminate areas.

Case fatality ratios vary widely between countries, with those countries with easily accessible and established health systems showing lower mortality rates compared to countries with limited facilities (Fig 2 and Table 4). In a previous review, untreated scrub typhus infection was associated with an estimated mortality of 6.0% (median, range 0–70.0%) [15]. This review of treated scrub typhus, which included 39 studies and 91,692 patients found a median mortality of 1.4% (range 0–33.3%).

**Table 4:** Overview of case fatality ratios reported for treated scrub typhus.

Date of study, Author	Country	No. with ST	CFR %	Comments
**2006–2012, Yang**	China	308	0	DALY study in Laiwu
**1995–2006, Liu**	China	480	0	Fever study
**2006–2014, Wu**	China	54,558	0.07	National surveillance data
**2012, Wei**	China	29	13.8	Outbreak investigation in a Guangzhou city park
**2002, Vaz**	India	12	0	Outbreak investigation in soldiers
**2014, Luthra**	India	197	1.0	Fever study
**2011, Gurung**	India	63	1.6	Fever study
**2008–2012, Thomas**	India	262	1.9	Fever study in children
**2010–2011, Mahajan**	India	253	5.1	Fever study
**2013, Masand**	India	30	6.6	Fever study
**2009–2010, Varghese**	India	154	7.8	Fever study
**2005–2010, Varghese**	India	623	9.0	Fever study
**2014, Khandelwal**	India	52	9.6	Fever study in children
**2007–2008, Chrispal**	India	189	12.2	Fever study
**2013–14, Sharma**	India	228	13.6	Fever study
**2011–2012, Kumar**	India	49	16.3	Fever study
**2014, Singh**	India	13	23.1	ICU patients with pulmonary complications
**2003–2004, Sharma**	India	52	28.8	Outbreak investigation Himachal Pradesh
**2009–2010, Narvencar**	India	15	33.3	Fever study
**2000, Matsui**	Japan	756	0.3	National surveillance data
**2007–2008, Yasunaga**	Japan	210	1.0	Retrospective use of discharge data
**2003–2012, Yoo**	South Korea	141	0	Hospital surveillance data
**1985–1990, Yi**	South Korea	189	0	Clinical diagnosis only
**2008–2012, Park**	South Korea	30,478	0.1	National surveillance data
**2004–2006, Kim**	South Korea	208	0.5	Case control study at single site
**2001–2011, Jang**	South Korea	771	1.4	Retrospective study in single site
**2000–2006, Lee**	South Korea	297	6.1	Retrospective study in single site
**2000–2006, Kim**	South Korea	160	6.3	Scrub typhus in patients with liver disease
**2003–2011, Dittrich**	Laos	31	13.6	Patients with CNS symptoms
**1948–1950, Bailey**	Malaysia	35	0	Early trials of antibiotic use
**2008, Premaratna**	Sri Lanka	26	0	Outbreak investigation in soldiers
**2006–2010, Wang**	Taiwan	126	0	Retrospective study in single site
**1952, Prezyman**	Taiwan	47	0	Early trial of antibiotic use
**1996, Watt**	Thailand	19	0	Prospective evaluation of antimicrobial response
**1985–2002, Silpapojakul**	Thailand	73	1.4	Case series of children
**2001–2002, Thap**	Thailand	18	16.7	Patients with septic shock
**2011–2012, Sriwongpan**	Thailand	257	13.6	Retrospective hospital series
**1965, Hazlett**	Vietnam	32	0	Case series from military hospital
**2001–2003, Nadjm**	Vietnam	251	1.2	Hospital surveillance data

### Disability Adjusted Life Year (DALY) data

The burden of disease data for scrub typhus is highly limited. Only one study, from Laiwu Province in China, has calculated the DALYs associated with scrub typhus [24]. This study estimated that 13 DALYs were lost due to scrub typhus across the province (6 in males, 7 in females at a rate of 1.06/100,000). However, in this province no deaths were reported and therefore these data cannot be extrapolated to countries such as India or Laos with evidence of scrub typhus associated mortality. A South Korean study evaluating the net benefit of a scrub typhus prevention program, estimated the cost of scrub typhus (medication and hospital costs and loss of earnings) at $6.6 million per year in 2008 [56]. However, scrub typhus mortality in South Korea was only 0.14% and 75% of patients with a diagnosis were hospitalized. Therefore, these figures cannot be applied to other economically poorer countries where health practice is very different [17].

## Discussion

Scrub typhus represents a major cause of treatable febrile illness across Asia, but its disease incidence remains elusive. Fever remains one of the major reasons to seek healthcare in tropical regions but their causes remain ill-defined [57]. Access to updated evidence on incidence and trends for common causes of febrile illnesses is essential for guiding and informing global, regional, and national health policies. This systematic review collated all currently available literature regarding the disease burden and economic impact of scrub typhus and the result is sobering; there are very few studies and they have great heterogeneity in methodology.

Acquisition of estimates for incidence and mortality proved difficult, as numerators had varying levels of confidence in diagnosis or denominators were either absent, or required further extrapolation. Ideally, data derived from population-based surveillance studies would be graded considerably higher than from hospital-based surveillance, but unfortunately no non-hospital-based surveillance data are publicly available for scrub typhus—unlike for diseases like typhoid where these data are readily available for various countries [58]. Further, the epidemiology of scrub typhus within a country is heterogeneous–the pronounced seasonality of these diseases and the changing urban/rural distribution, with defined areas of high infected mite intensities (mite islands) challenge the common approaches of disease incidence evaluation [59, 60]. Febrile illness surveillance should be performed in multiple representative areas, ideally covering one full calendar year before inferences on national disease incidence can be made [58].

### Incidence surveillance

Only 5 countries have established scrub typhus surveillance systems. All of these have shown an increasing minimum incidence of scrub typhus over recent years, with increasing evidence of shift towards urbanized areas. However, the apparent increase in minimum incidence is confounded by local enhanced knowledge of the disease and it remains uncertain whether these data reflect true de novo emerging disease or emerging awareness of a pre-existing disease. Surveillance systems also use diverse diagnostic tests and therefore inter-country comparisons are not always possible. There are no data on whether these surveillance systems have been evaluated to determine an estimate of missed cases, however it is likely that the numbers are conservative estimates. Regardless of these flaws, surveillance systems are an essential part of disease control strategies. Improved febrile disease surveillance providing national data should be initiated in more afflicted countries, as this would result in morbidity and mortality data that could be used to direct healthcare resources, future vaccine demand and delivery and assessment of effectiveness of any control programs.

Clearly, striving towards improved surveillance should be key, with a focus on providing reliable numerators (using diagnostic assays with suitable sensitivities and specificities), and representative denominators (well-defined target populations). Additionally, no ‘multiplier data’ or ‘multiplier studies’ are available—these are considered to improve estimation of incidence by using healthcare utilization surveys and to correct for under-ascertainment in healthcare facility studies [58].

### Seroprevalence

Seroprevalence data was available from 5 countries only–indicating high background exposure levels, and therefore a high probability that larger numbers of unidentified and/or asymptomatic infections occur. Disease seroprevalence data must be interpreted with caution due to unknown antibody dynamics over time and uncertainty as to whether those seropositive became sick or were asymptomatic. In scrub typhus, both humoral and cell-mediated protective immune responses wane over time, but detailed understanding of this remains elusive [61]. Moreover, the population-wide frequencies of patients with reversion to seronegativity and potential disease susceptibility remain unknown, and therefore the actual exposure in these studies is likely to be substantially higher [62].

### Fever studies

Scrub typhus is a leading cause of treatable non-malarial febrile illness in prospective fever etiology studies (n = 14). An increasing number of studies have unraveled the major contribution of scrub typhus to the febrile illness burden. However, the large variation of scrub typhus rates in prospective fever studies (median 23.4% IQR 5.2–39.7 ranging from 1–96.9% depending on country and patient selection), reflect a lack of standardization and comparability among study designs and diagnostic modalities used. None of these studies have used modeling or extrapolation to take into account data from healthcare utilization surveys, which may give a more accurate idea of numbers of people with scrub typhus. In addition, recent studies have raised concern on the persistence of *O*. *tsutsugamushi* after treatment, especially using bacteriostatic drugs such as tetracyclines and macrolides [63, 64].

### Vulnerable populations and case fatality ratios

Based on very limited data, scrub typhus is likely to have considerable impact on vulnerable populations–the median untreated mortality of scrub typhus in the elderly was ~29%—approximately 5-fold higher compared to the overall population mortality of 6% [15]. In women with scrub typhus during pregnancy, miscarriages occurred in 17% and poor neonatal outcomes in 42% of cases, which is more severe than the consequences of malaria in pregnancy [65]. Further, the mortality in patients requiring a lumbar puncture for scrub typhus CNS complications in Laos was 14% [59]. Scrub typhus is usually an easily treatable disease and the majority of these complications could be prevented by early recognition/diagnosis and increased usage of empirical doxycycline [66].

It is difficult to draw any definitive conclusions from the case-fatality data due to the heterogeneity in studies. They range from national surveillance data to case series of those admitted to ICU. National surveillance data from China, Japan and Korea provide *case fatality ratios* of 0.068–0.26%. However, the health facilities in these countries are significantly more advanced than other endemic countries. The fever studies from South India provide estimates of case fatality risk, but they vary from 0–33.3%—importantly, these data included patients who presented to hospital and therefore will miss those that do not have severe disease.

### Health economics

DALY data are lacking in all countries except from one area of China, where a rate of 1.06/100,000 people was found, with a zero mortality rate. Case series and studies from Taiwan and India examining long-term complications, imply that the mortality and morbidity from scrub typhus is under-recognized and that possible long term consequences may occur many years later, and may be important contributors to the overall DALY burden [37, 67]. Despite scrub typhus being the foremost cause of treatable febrile illness in Asia it is not evaluated by the Global Burden of Disease studies [68].

### Study limitations

This study involved an extensive search of the literature and includes up-to-date and relevant studies. However, there are several limitations; as English is not the native language in the majority of countries where scrub typhus is endemic, there is a potential bulk of relevant literature that is not indexed in the databases used. The risks of publication bias and the heterogeneity of methods and reporting in the articles limit the conclusions. Specific difficulties relating to the diagnosis of scrub typhus suggest that studies reporting data from national surveillance systems are likely to suffer from missing data due to those that do not seek medical attention are misdiagnosed or not reported. The majority of fever studies suffers from selection bias and often relies on suboptimal diagnostic tools.

### Future focus

Reports from Africa, the Middle East and most recently South America, suggest that scrub typhus is more widespread than previously appreciated. The molecular detection of *Orientia* spp. in rodents from Southern France and Senegal suggest that rodent-mite cycles could maintain the pathogen in nature but whether these *Orientia* spp. represent human pathogens is unknown [69]. The countries most affected by scrub typhus are currently experiencing profound demographic, economic and ecological changes [70]. Deforestation, growing cities and climate change may lead to migration of rodents carrying infected mites and expand to more urban and non-endemic areas [8, 11, 16]. Recently the impact of an earthquake on exposing the population to the possibly perturbed soil dwelling vectors causing scrub typhus was highlighted in Nepal [71].

Ancestor *et al*. mapped non-malarial causes of fever, including scrub typhus, in the Mekong region [2]. Kelly *et al*. developed a vector map of scrub typhus based on literature review to include probable and confirmed cases that included geo-referenced locations [72]. These are useful resources that can be built upon to estimate incidence in areas where data is limited. In scrub typhus the extracted information of studies from the 1940s requires careful consideration to identify what data are clinically relevant today. Derne *et al*. summarized and mapped the distribution of rickettsia and their vectors in Oceania, confirming the widespread presence and providing a scaffold to build upon [73]. Ideally, concerted efforts in providing well maintained up-to-date mapping of human cases and vector (chigger mite) distribution would contribute substantially to understanding the burden of disease.

Burden of disease studies often use syndromic ‘envelopes’ for certain conditions (for example “diarrhea” or “fever”). Developing a fever ‘envelope’ approach for estimating its burden of disease, in conjunction with detailed fever etiology studies would provide improved, standardized and globally comparable incidence data [74, 75]. The resulting data could be stratified further and would inform on the actual burden of disease, as well as provide valuable baseline data to support economic evaluations and mathematical modeling of future interventions [76]. For example, an incentive for identifying endemic areas of scrub typhus may result in increasing cost-effectiveness of rapid diagnostic test (RDT) use. Testing for frequent bacterial pathogens is likely to be economical, reducing hospitalization rates, and informs not only treatment requirements, but also appropriate antibiotic usage [77].

In the case of dengue, the quality of data available has improved substantially and in 2010 there were an estimated 96 million apparent and 294 million unapparent dengue infections globally [78]. Although dengue and scrub typhus both top the list of fever etiologies in multiple studies in Asia, the more easily-treatable disease is neglected–it is time for more integrated expert collaborative research to provide these urgently needed objective data [57, 78, 79].

These data–despite their limitations–make a case for scrub typhus as an important neglected tropical disease of mainly rural populations, with an increasing urban proportion. In countries with established surveillance systems, the reported incidence is increasing and robust documentation of scrub typhus in Chile suggests a much wider global presence than previously understood. The lack of data on global incidence and disease burden highlights the need for this treatable infection to receive increased attention and research to inform health policy.

## Supporting information

S1 File PRISMA checklist. (DOC)
